# Insecticidal and biochemical effects of *Dillenia indica* L. leaves against three major stored grain insect pests

**DOI:** 10.3389/fpls.2023.1135946

**Published:** 2023-02-20

**Authors:** Kabrambam D. Singh, Arunkumar S. Koijam, Rupjyoti Bharali, Yallappa Rajashekar

**Affiliations:** ^1^ Insect Bioresource Laboratory, Animal Bioresources Programme, Institute of Bioresources and Sustainable Development, Department of Biotechnology, Government of India, Imphal, Manipur, India; ^2^ Department of Biotechnology, Gauhati University, Guwahati, Assam, India

**Keywords:** *Dillenia indica* L., biofumigant, stored grain pests, acetylcholinesterase, antioxidant enzyme system

## Abstract

The Last four decades have witnessed the banning of several synthetic insecticides mainly due to the development of resistance to the target pests and due to hazardous effects on humans and the environment. Hence, the development of a potent insecticide with biodegradable and eco-friendly nature is the need of the hour. In the present study, the fumigant property, and biochemical effects of *Dillenia indica* L. (Dilleniaceae) were studied against three coleopterans stored-products insects. The bioactive enriched fraction (sub-fraction-III) was isolated from ethyl acetate extracts of *D. indica* leaves and found toxic to rice weevil, *Sitophilus oryzae* (L.) (Coleoptera); lesser grain borer *Rhyzopertha dominica* (L.) (Coleoptera) and red flour beetle, *Tribolium castaneum* (Herbst.) (Coleoptera) with the LC_50_ values of 101.887, 189.908 and 115.1 µg/L respectively after 24 h exposure. The enriched fraction was found to inhibit the function of acetylcholinesterase (AChE) enzyme when tested against *S. oryzae, T. castaneum*, and *R. dominica* with LC_50_ value of 88.57 µg/ml, 97.07 µg/ml, and 66.31 µg/ml respectively, in *in-vitro* condition. It was also found that the enriched fraction caused a significant oxidative imbalance in the antioxidative enzyme system such as superoxide dismutase, catalase, DPPH (2,2-diphenyl-1-picrylhydrazyl), and glutathione-S-transferase (GST). GCMS analysis of the enriched fraction indicates three major compounds namely, 6-Hydroxy-4,4,7a-trimethyl-5,6,7,7a-tetrahydrobenzofuran-2(4H)-one, 1,2-Benzisothiazol-3(2H)-one, and Benzothiazole, 2-(2-hydroxyethylthio)-. Finally, we concluded that the enriched fraction of *D. indica* has insecticidal properties and the toxicity may be due to the inhibition of the AChE enzyme in association with oxidative imbalance created on the insect’s antioxidant enzyme systems.

## 1 Introduction

Every year there is a loss of 5-30 percent of the world’s total agricultural food production due to insect infestation on food grains (Rajashekar et al., 2010). The stored grain insects are known to inflict huge damage to stored grains and pulses through the consumption of kernels or accretion of exuviae, webbing, and cadavers (Rajashekar et al., 2012). *Sitophilus oryzae* (L.) (Coleoptera), commonly called rice weevil, and *Rhyzopertha dominica* (L.) (Coleoptera) (common name lesser grain borer), are two of the many primary pests which cause severe global economic losses while red flour beetle, *Tribolium castaneum* (Herbst.) (Coleoptera), is one of the secondary pests that inflicts damage to stored grain pests in many parts of the world. Generally, chemical-based fumigants are widely used to control the damage caused by insects. But it also brings along several shortcomings such as toxicity to humans and livestock, as well as other non-target organisms, secondary pest outbreaks, pest resurgence, adulteration of food products due to indiscriminate use, erratic supplies, and unavailability at critical periods, high price while also causing several environmental hazards such as ozone depletion. Persistent use of such chemicals also leads to the emergence of resistant strains of the targeted pests (Abubakar et al., 2020). Excessive exposure to chemical pesticides could cause oxidative stress to the human being that ultimately leads to many neurodegenerative diseases such as Parkinson’s disease, Alzheimer’s disease, etc. (Huang et al., 2016; Nandipati and Litvan, 2016; Sabarwal et al., 2018). For instance, methyl bromide (now banned) was an effective fumigant used for neutralizing insects on soil and storage structures. Many studies have indicated that prolonged exposure to it has a high effect on the human central nervous system (de Souza et al, 2013; Park et al., 2020). Considering the problems, scientists all over the world are constantly exploring for a safer source for developing eco-friendly bioinsecticides. Plants being one of the richest sources of bioactive molecules may provide potential alternatives to currently used chemical-based approaches (Rajashekar et al., 2012; Singh et al., 2021). Available literature highlights the use of plant-derived botanicals as a source for new insecticides (Miresmailli and Isman, 2014; Rajashekar et al., 2016; Devi et al., 2020; Singh et al., 2021). Therefore, there is a great scope for botanical insecticidal compounds. Providing the best quality seeds for cultivation will enhance productivity thereby providing the best economic and social return. Insect infestation is one of the major factors that affect the viability of seeds meant for prolonged storage. Sometimes the insecticides used to control the stored insects also hamper seed germination. Such things need to be taken care of before deciding the class and dose of the insecticide to be used.

*Dillenia indica* L. (elephant apple), is a perennial middle-size tree found in tropical, subtropical, and temperate zones. The genus ‘*Dillenia*’ spreads from Madagascar to Fiji Island, and from there it is distributed to Northern and Southern Himalayan slopes, and Southwestern China (Hoogland, 1952). In India, this tree is distributed in the sub-Himalayan tracts, West Bengal, Madhya Pradesh, Assam, North-Eastern India, and South Indian States. This plant has several important biological activities including insecticidal properties (Reddy et al., 2010). They are known to have antidiabetic, antioxidant activity, anti-inflammatory, as well as anticancer properties (Barua et al., 2018). Some literature reported that the spreading of *D. indica* leaves over the stored rice repelled rice weevil (*S. oryzae*) (Bhattacharjee and Ray, 2010). No scientific validation has been provided till date in this aspect. The present study tried to explore the potential insecticidal property of the plant-derived product along with its effect on the antioxidant enzyme system. The study also intended to analyze the phytochemical composition of the bioactive fraction responsible for the fumigant activity. Further, the possible mode of action mechanism was studied with respect to the inhibitory effect on acetylcholinesterase enzyme.

## 2 Materials and methods

### 2.1 Collection and preparation of sample

The fresh and matured leaves of *D. indica* were collected from Imphal West, Manipur (N24°49.258’, E093°56.411’) and authenticated by Dr. Biseshwori Thongam, Scientist-E (Taxonomist), Institute of Bioresources and Sustainable Development, Imphal, Manipur, with voucher number IBSD/M-284. They were properly washed and semi-dried in shade for 4-5 days. The samples were finely powdered using an electric grinder and packed in air-tight poly bags for further use.

### 2.2 Chemicals

Pyrogallol, Catalase, reduced glutathione (GSH), 2,2- diphenyl-1-picrylhydrazyl (DPPH), 1-chloro-2,4-dinitrobenzene (CDNB), Acetylthiocholine chloride, and 5,5-dithio-bis-2-nitrobenzoic acid (DTNB) were procured from Sigma Chemical Co. (St. Louis, MO, USA); hydrogen peroxide, sodium hydroxide, sodium di-hydrogen phosphate, L-ascorbic acid, and sodium carbonate were obtained from Sisco Research Laboratory, Mumbai, India. Sodium Chloride, Magnesium Chloride, and Tris-base were purchased from Himedia Laboratories, Mumbai.

### 2.3 Extraction and isolation of bioactive enriched fraction

**Figure 1:**
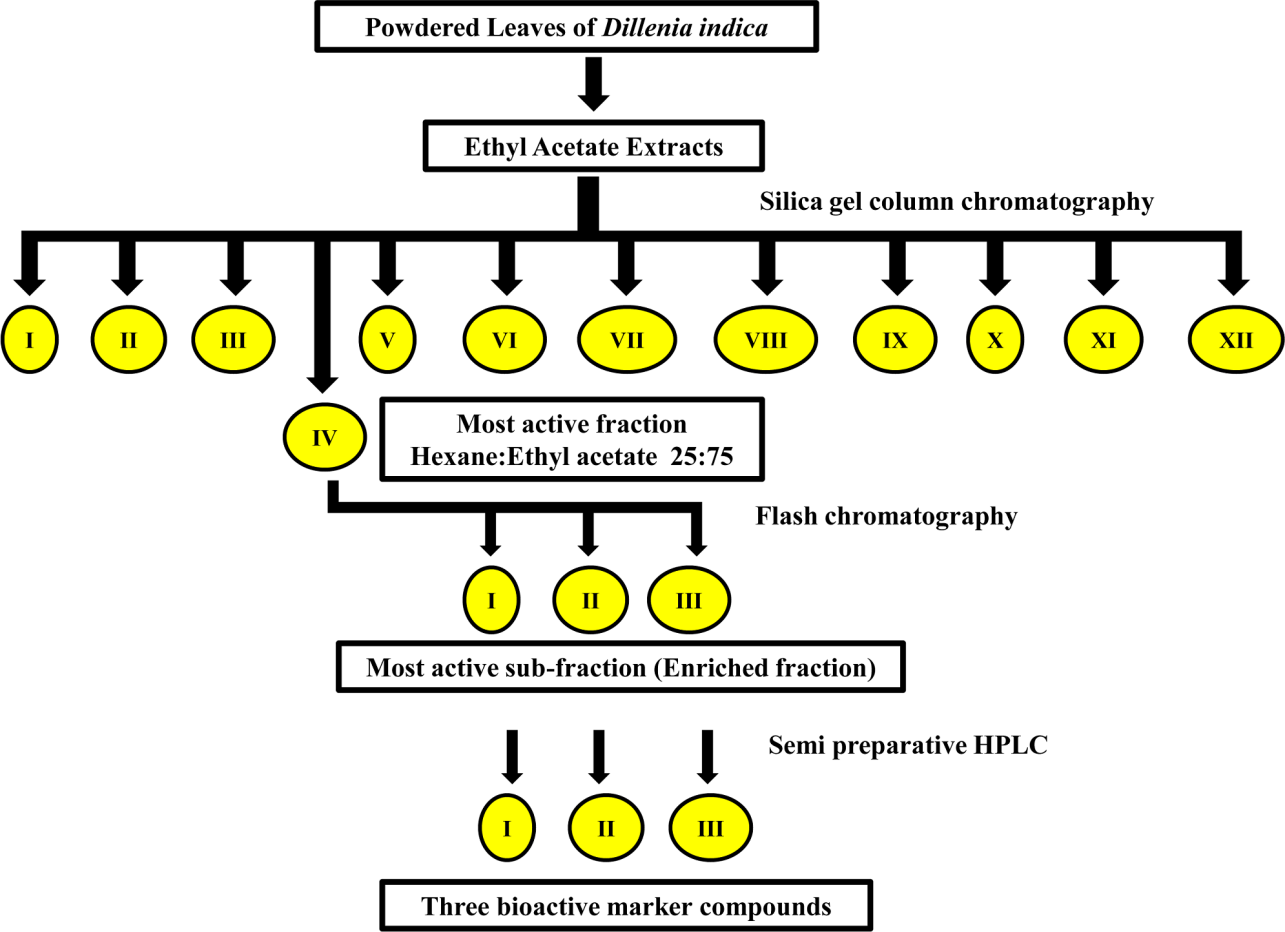
Schematic diagram of the isolation of bioactive enriched fraction from ethyl acetate extract of *D. indica* leaves.

One kilogram of powdered leaves samples was used for the sequential extraction of phytochemicals in the Soxhlet apparatus. The extraction was done for 8-9 h using different solvents of increasing polarity viz., hexane, petroleum ether, ethyl acetate, chloroform, acetone, and methanol. The solvent extracts were filtered with Whatman paper No. 1 and solvents were evaporated using a rotary vacuum evaporator {Rotavapor R100 (Buchi) Switzerland} under low pressure, at a temperature of 45°C. Each extract was tested for fumigant properties against three stored product insects, viz., *S. oryzae, T. castaneum*, and *R. dominica*. Extract with the highest mortality was further subjected to bioassay-guided isolation of the bioactive enriched fraction using several chromatographic techniques. Silica gel column chromatography with mesh size 60-120 mesh and glass column of 50 cm length and 3 cm diameter was used for the separation of phytochemicals. The active extract was first eluted with 100% hexane, followed by hexane and ethyl acetate mixture, ethyl acetate, and acetone mixture, and then acetone and methanol mixture at different ratios (75:25; 50:50; 25:75; 0:100). Solvents from all 13 fractions were evaporated under reduced pressure and the residue was dissolved in a known volume of acetone. These solutions were tested for fumigation activity against the three stored product insects. Fractions showing the highest fumigant activity were pooled and subjected to Flash chromatography (CombiFlash Rf^+^ Lumen, Teledyne ISCO, USA) with solvent system hexane and ethyl acetate and 0.5% methanol as a modifier, for further separation of bioactive compounds. The eluted sub-fractions were again tested for fumigation activity against the test insects. The most active enriched fraction based on corrected mortality (Sub-Fraction-III) (**Figure 1**) was collected and used for further experimental purposes. The enriched fraction was further subjected to semi-preparative high performance liquid chromatography (HPLC) for purification and characterization of the bioactive marker compound(s).

### 2.4 GCMS analysis of the bioactive marker compounds

The marker compounds isolated from the enriched fraction were identified by Gas Chromatography Mass Spectrometry (Thermo Scientific Trace 1300 Gas Chromatograph & TSQ 8000 DUO Mass Spectrometry) having Quadrapole detector analysis. GC detection was performed at the ionization energy 70eV. The injector and mass transfer line were set at 250°C and 280°C. the carrier gas used was helium at the flow rate of 1ml/min and the injection volume was set at 0.5 μl. The initial column temperature was programmed from 40°C for 1 min to 250°C at a rate of 5°C/min heating ramp and then held at 250°C for 20 min. The compounds were identified using the National Institute of Standards and Technology (NIST) library 2017 based on the comparison of their mass spectra with that of the library.

### 2.5 Fumigant toxicity

Fumigant toxicity assays were performed following the methodology from Rajashekar et al. (2016). Twenty adults of both sexes of *S. oryzae, T. castaneum*, and *R. dominica* were separately released inside the fumigation chamber of one liter volume capacity. Each chamber was infused with different leaf extract solutions at a fixed concentration of 50 mg/L air to a filter paper already placed inside the chamber. The extract solutions were injected using a Hamilton syringe through a rubber septum fitted to the chamber’s lid and the infused filter papers were placed on the under surface of the glass chambers which were checked from direct contact with the insects. An equal volume of pure acetone was used as solvent control. The number of dead insects was determined after 24 h. Dose-response relationship was done for the most active enriched fraction with concentration ranges from 50 to 400 μg/L air. The percentage of corrected mortality was calculated using the Abbott formula equation (Abbott, 1925).

### 2.6 *In vitro* acetylcholinesterase activity assay

The effect of the enriched fraction on the insect’s acetylcholinesterase enzyme (AChE) was studied following Ellman’s method with slight modification (Ellman, 1959). The AChE enzyme hydrolyses the substrate acetylthiocholine to produce acetate and thiocholine. Thiocholine reacts with Ellman’s reagent (DTNB) to produce 2-nitrobenzoate-5-mercaptothiocholine and 5-thio-2-nitrobenzoate which can be detected at 412 nm. The enzyme activity was tested against crude enzyme extract of *S. oryzae, R. dominica*, and *T. castaneum in -vitro* conditions. The insects (20 adults each) were homogenized using 0.5M Tris-HCl buffer and stored at -20^0^C. For the study, crude enzyme extract was pre-incubated with the enriched fraction and with standard inhibitor (Pyridostigmine bromide) at different doses of 25, 50, 75, and 100 μg/ml of insect’s enzyme extract at 37°C for 30 mins. A microplate reader was used to measure the difference in the absorbance. In a microplate well 200 µl of the reaction mixture, 3 µl of 0.1M acetylthiocholine chloride, 10 µl of insect homogenate, and 87 µl of water were added to make the total volume of 300 µl. The reaction mixture is prepared by adding 10.5 ml of cocktail (13 ml of 1M NaCl, 2 ml 1M MgCl_2_, 10 ml of 0.5M Tris-HCl, and 10 ml of 0.2M EDTA), 3 ml of 1mM DTNB and 6.5 ml of water in a reagent bottle. The reaction is initiated either by adding the treated enzyme or substrate and expressed as percentage inhibition.

Inhibition (%) = 100 - Change of sample absorbance/Change of blank absorbance X 100

### 2.7 Antioxidant enzymes

#### 2.7.1 Superoxide dismutase activity

The pyrogallol (2mM) autooxidation method described by Marklund and Marklund (1974) was followed for measurement of SOD activity in the tested insects, *S. oryzae* and *T. castaneum*. The reaction mixture contained 2 mM pyrogallol in 0.1M Tris buffer (pH 8.2) and the enzyme. The addition of substrate in the reaction mixture started the reaction and the absorbance was read at 420 nm for 3 min at an interval of 1 min. The SOD activity was expressed as enzyme units/mg protein. The amount of enzyme that inhibits auto-oxidation by 50% is referred to as one unit of enzyme activity.

#### 2.7.2 Catalase activity

The protocol given by Aebi (1983) was used to assay the catalase activity in the tested insects. The reaction mixture contained 3% H_2_O_2_ in 0.05M phosphate buffer (pH 7.0). The reaction was started by the addition of enzymes and the change in the absorbance at 240 nm was read for 3 min and the activity was expressed as µmole H_2_O_2_/min/mg protein.

#### 2.7.3 Glutathione-S-transferase

Glutathione S-transferase (GST) activity was measured following the method of Warholm et al. (1985) with CDNB as the substrate. The reaction mixture contains 20 mM GSH and the enzyme (supernatant) in 0.1M phosphate buffer (pH 7.4). The reaction was started by adding 30 mM CDNB and the change in absorbance at 344 nm was monitored in a UV-visible spectrophotometer. The enzyme activity was expressed as µmole CDNB conjugate/min/mg protein.

#### 2.7.4 DPPH radical scavenging assay

The protocol given by Yamaguchi et al. (1998) was used to measure the DPPH radical scavenging activity in *in-vitro* conditions. Briefly, 1 ml of 0.1 mM DPPH solution in 95% ethanol was treated with different concentrations of the active enriched fraction, shaken, and incubated at room temperature for 20 min, and the absorbance was read at 517 nm against a blank. Ascorbic acid was used as the standard to compare the inhibition ability of the enriched fraction to that of the standard. The radical scavenging activity was calculated using the following equation:


Scavenging effect (%) = [1− A Sample (517nm) /A Control (517nm)] x 100

Total protein content of the sample was measured by the method of Lowry et al. (1951) using BSA as the standard.

#### 2.7.5 Seed germination test

Wheat, *Triticum aestivum* L., and green gram, *Vigna radiata* (L.) R. Wilczek seeds were surface sterilized using 1% sodium hypochlorite for 10 minutes and washed properly with autoclaved distilled water. The 50 sterilized seeds of wheat and green gram were separately kept on Whatman filter paper no. 1 already treated with 100 mg/L and 500 mg/L of enriched fraction and placed in glass petri plates (90X15 mm, borosil, India). The filter paper was kept moist throughout the experimental period by spraying it with distilled water. The germination test was performed for 48 h and 5 days. In the control petri plates, the Whatman filter paper was only soaked with sterilized distilled water. The observation was taken after 48 h and 5 days and the germination percentage was calculated (Rajashekar et al., 2016).

### 2.8 Data analysis

LC_50_ values were determined using Probit analysis (Finney, 1971) and Statplus 2007 software and computer program SAS (version 6.12, SAS Institute Inc. Cory, NC, USA) were used to analyze the data using One-Way ANOVA (p<0.05) by Newman-Keuls test.

## 3 Result

### 3.1 Fumigant toxicity test of extracts and enriched fraction

**Figure 2:**
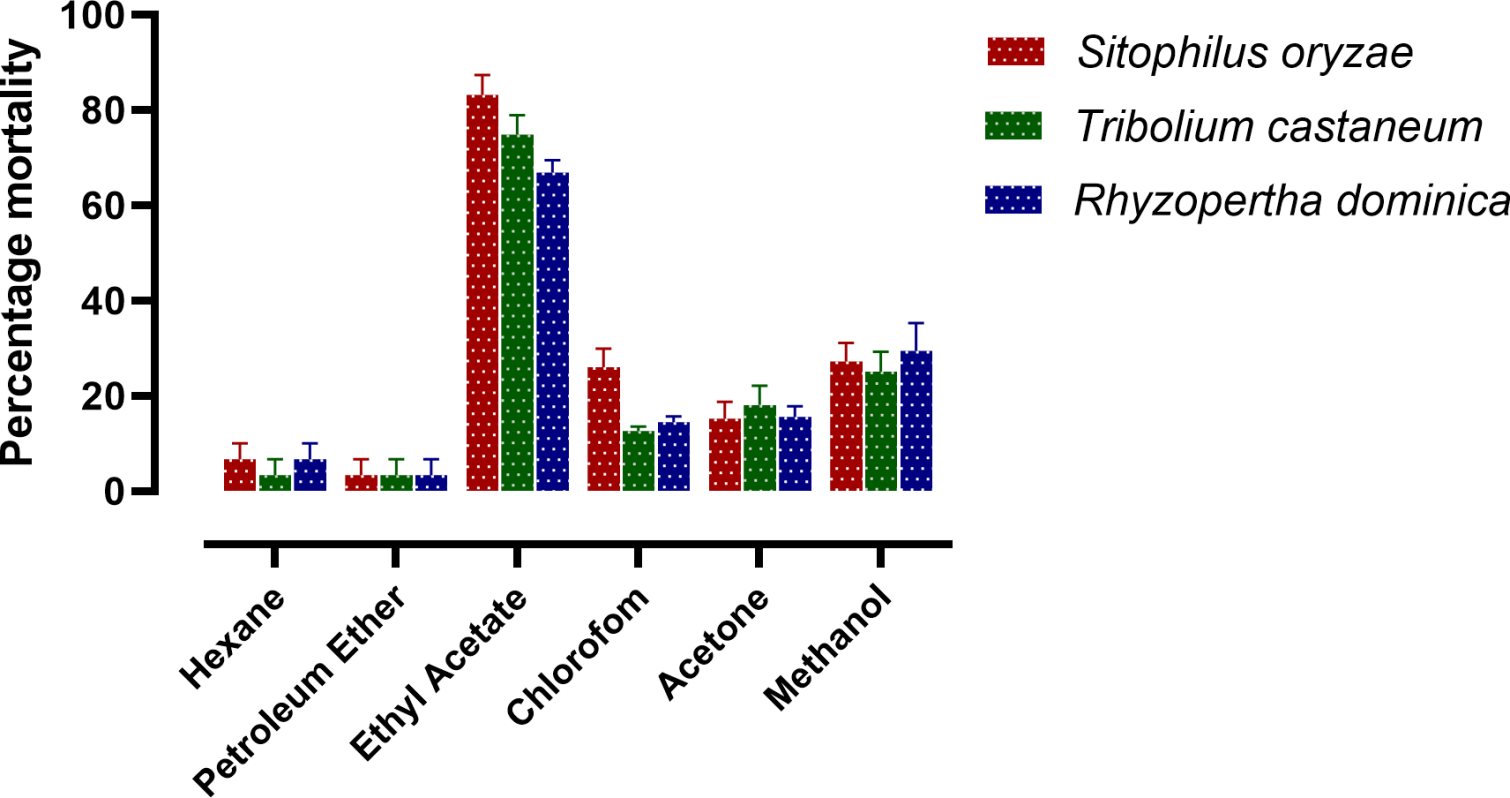
Fumigant toxicity of different extracts from *D. indica* leaves, at 50 mg/L, against *S. oryzae, T. castaneum* and *R. dominica*. (n= 4).

Experimental results reveal that among all the extracts ethyl acetate extract of *D. indica* leaves showed maximum fumigant activities against *S. oryzae, T. castaneum*, and *R. dominica* (**Figure 2**). **Table 1** shows that the bioactive enriched fraction isolated from ethyl acetate extract exhibited toxicity to *S. oryzae, T. castaneum*, and *R. dominica* with the LC_50_ values of 101.88, 198.89, and 115.1 µg/L air respectively after 24 h exposure.

**Table 1:** Insecticidal activity of the enriched fraction isolated from ethyl acetate extract of *D. indica* leaves against *S. oryzae, T. castaneum* and *R. dominica* by using fumigant assay method. (n= 4).

Insects	LC_50_ value ^a,b^	Slope ± SE	Chi square	Degree of freedom
*S. oryzae*	101.88 ± 10.46	0.1446 ± 0.029 (0.077 – 0.211)	1.22	3
*T. castaneum*	198.89 ± 15.9	0.1674 ± 0.020 (0.12 – 0.21)	1.67	3
*R. dominica*	115.1 ± 8.63	0.148± 0.023 (0.096 – 0.2)	1.25	3

a LC_50_= µg/L air.

b Values in parenthesis represent conﬁdence limits by probit analysis (Finney, 1971), n=4.

### 3.2 Compound identification by GCMS

**Figure 3:**
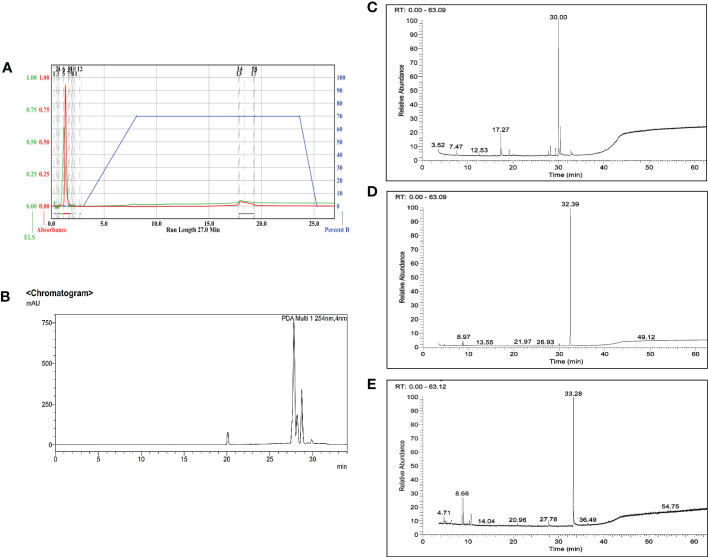
Isolation and characterization of the bioactive markers from the enriched fraction **(A)**. Chromatogram from Flash chromatography, **(B)**. Chromatogram from Semi- preparative HPLC, and **(C–E)** Representative GC-MS ion chromatogram of the three isolated bioactive compounds, 6-Hydroxy-4,4,7a-trimethyl-5,6,7,7a-tetrahydrobenzofuran-2(4H)-one (commonly known as loliolide), 1,2-Benzisothiazol-3(2H)-one, and Benzothiazole,2-(2-hydroxyethylthio)- respectively.

Three major bioactive compounds were separated and eluted from the enriched fraction using semi-preparative HPLC. Gas chromatography mass-spectrometry analysis identified the isolated bioactive as 6-Hydroxy-4,4,7a-trimethyl-5,6,7,7a-tetrahydrobenzofuran-2(4H)-one (IUPAC name: 6-hydroxy-4,4,7a-trimethyl-6,7-dihydro-5H-1-benzofuran-2-one), 1,2-Benzisothiazol-3(2H)-one (IUPAC name: 1,2-benzothiazol-3-one), and Benzothiazole,2-(2-hydroxyethylthio)- (IUPAC name: 2-(1,3-benzothiazol-2-ylsulfanyl)ethanol) with reverse search index (RSI) value of 905, 861, and 941 respectively (**Figure 3**).

### 3.3 *In - vitro* inhibition of AChE enzyme

In an *in - vitro* study, we investigated the influence of the bioactive enriched fraction on the insect’s acetylcholinesterase enzyme (AChE). The enriched fraction was found to significantly inhibit the AChE activity of *S. oryzae, T. castaneum*, and *R. dominica*. **Table 2** reveals various percentage inhibitions of the AChE enzyme in different doses of the enriched fraction. The percentage inhibition of different doses of the enriched fraction was compared with Pyridostigmine (standard AChE inhibitor) at equal concentration. The percentage inhibition value ranges from 20.69% to 55.17%, 12.86% to 51.42%, and 26.28% to 66.67% for different doses with IC_50_ values of 88.57 µg/ml, 97.07 µg/ml, and 66.31 µg/ml on *S. oryzae, T. castaneum*, and *R. dominica*, respectively.

**Table 2:** Percentage inhibition of enriched fraction on AChE enzyme activity.

Concentration (μg/ml)	Percentage inhibition of AChE enzyme					
	*S. oryzae*		*T. castaneum*		*R. dominica*	
	Pyridostigmine	Enriched fraction	Pyridostigmine	Enriched fraction	Pyridostigmine	Enriched fraction
25 μg/ml	58.04 ± 1.1^a^	20.69 ± 1.7^a^	48.57 ± 2.4^a^	12.86 ± 1.4^a^	56.41 ± 2.5^a^	26.28 ± 2.8^a^
50 μg/ml	72.99 ± 2.3^b^	29.89 ± 2.3^b^	65.71 ± 1.9^b^	30 ± 3.8^b^	66.67 ± 1.2^b^	37.18 ± 1.3^b^
75 μg/ml	83.33 ± 2.0^c^	45.4 ± 1.2^c^	72.85 ± 1.4^c^	41.43 ± 1.4^c^	79.48 ± 1.2^c^	62.82 ± 2.7^c^
100 μg/ml	94.02 ± 1.0^d^	55.17 ± 1.5^d^	88.57 ± 2.8^d^	51.42 ± 1.4^d^	92.30 ± 1.0^d^	66.67 ± 2.6^d^

Data are given as Mean ± SEM (n=3). Values followed by different letters within the vertical columns are signiﬁcantly different (P< 0.05) by Duncan’s multiple range test.

### 3.4 Effect of the enriched fraction on insect’s antioxidant enzyme systems

**Figure 4:**
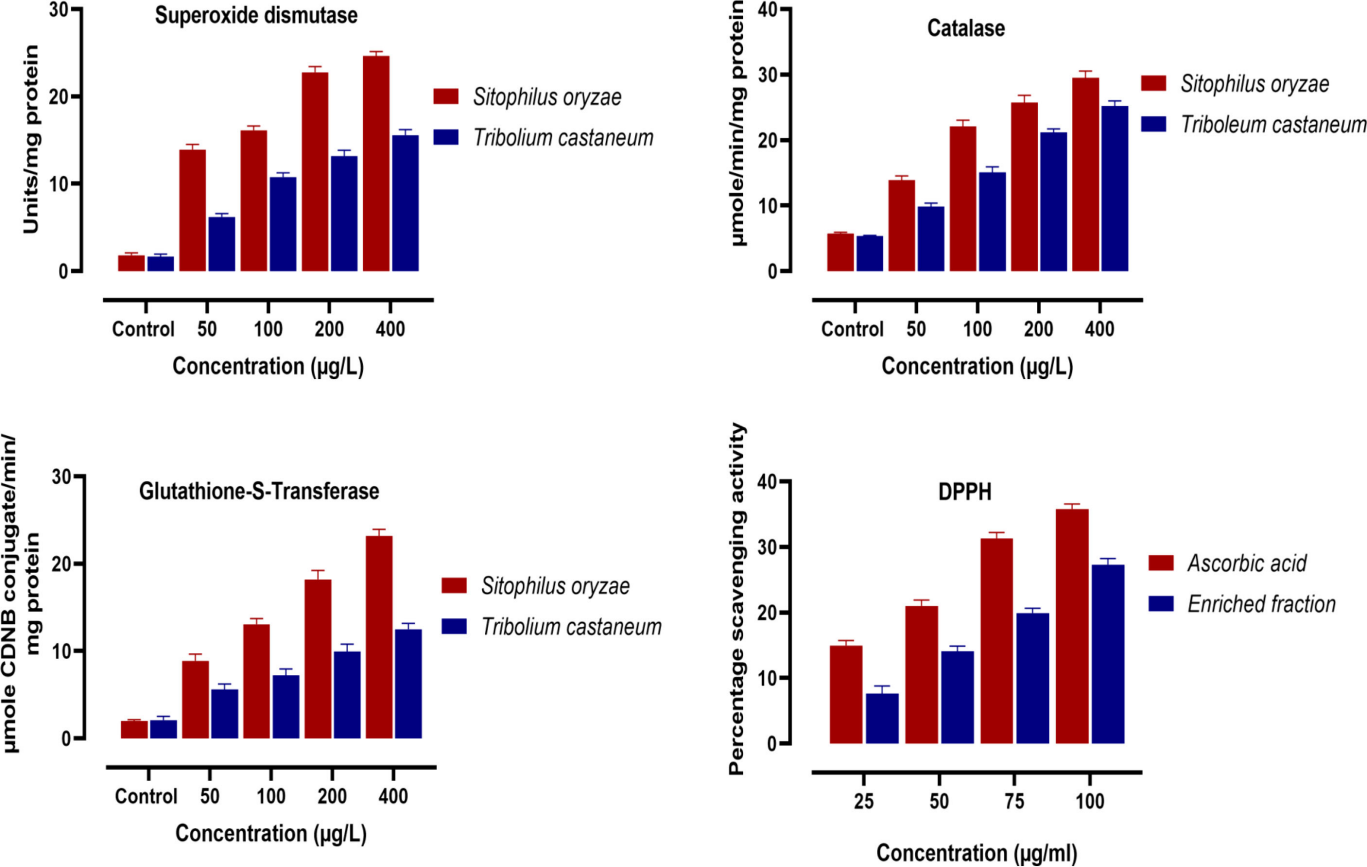
Effect of different concentrations of the enrich fraction eluted from ethyl acetate extract of *D. indica* leaves on the activity of enzymatic antioxidants in *S. oryzae*, and *T. castaneum*. (n=4).

In the present study, the effect of the enriched fraction on the activities of antioxidant enzymes in the tested insects, viz., *S. oryzae* and *T. castaneum*, were estimated. **Figure 4** indicates that the active enriched fraction caused significant impairment in the enzymatic (SOD, Catalase, GST) as well as non-enzymatic (DPPH) antioxidant systems of both *S. oryzae*, and *T. castaneum*. The results showed a significant increase in the activity of SOD, Catalase, and GST. The percentage inhibition of the scavenging activity of DPPH was also found to increase as we increase the concentration of the active enriched fraction (**Figure 4**).

### 3.5 Seed germination test

The effect of the bioactive enriched fraction on percentage germination of wheat and green gram seeds were determined. It was found that both treatments i.e., 100 and 500 mg/l have no significant effect on the seed germination at different exposure periods. The percentage of seed germination ranged from 96% to 98.67% and 94.33% to 98.67% at different concentration and exposure time on both wheat and green gram, respectively (**Table 3**).

**Table 3:** Effect of the enriched fraction on seed germination of wheat and green gram.

Dosage (mg/l)	Percentage seed germination			
	48 h		5 d	
	Wheat	Green gram	Wheat	Green gram
100	97.3 ± 0.6^a^	95.33 ± 1.3^a^	98.67 ± 1.2^a^	98.67 ± 1.3^a^
500	96 ± 1.2^a^	94.33 ± 1.2^a^	96.67 ± 0.6a	97.33 ± 1.7^a^
Control	99.33 ± 0.6^a^	98.67 ± 0.6^a^	99.33 ± 0.6^a^	98.67 ± 1.3^a^

Data are given as Mean ± SEM (n=4). Values followed by same letter within the vertical columns are not signiﬁcantly different (P< 0.05) by Duncan’s multiple range test.

## 4 Discussion

Medicinal and aromatic plants are good sources for many biological activities. Several plant species are known to contain phytochemicals that can be used as insecticides (Rajendran and Sriranjini, 2008; Green et al., 2015; Devi et al., 2021). Natural insecticides are preferable to synthetic chemical-based insecticides because of their eco-friendly nature (Rajashekar et al., 2016; Samada and Tambunan, 2020). Though many plants have been studied for their insecticidal property, there are several more with huge potential yet unexplored. *D. indica* has several important biological properties as compiled and presented by Barua et al. (2018). But no significant scientific research has been done regarding its insecticidal property despite the claims that the plant is traditionally used by farmers to control stored grain pests in the Northeastern parts of India (Bhattacharjee and Ray, 2010). In the present study, we tried to provide scientific validation to those traditional approaches.

The present study revealed that ethyl acetate extract of *D. indica* leaves has maximum fumigant toxicity against *S. oryzae, T. castaneum*, and *R. dominica*. The LC_50_ values of most active enriched fraction were relatively lower than methyl bromide (MeBr), which is a commercially available grain fumigant, with LC_50_ values of 0.67 mg/L and 1.75 mg/L against *Sitophilus zeamais* Motschulsky and *T. castaneum* adults respectively (Liu and Ho, 1999). Several similar experiments have been performed on different plants. In a study, Rajashekar et al., 2012 reveal that Coumaran, a natural fumigant isolated from the methanolic extract of leaves of *Lantana camara* (Verbenaceae) has fumigant toxicity against *S. oryzae*, *Callosobruchus chinensis* (Fab.) and *T. castaneum* with LC_50_ values of 0.45 µg/L, 0.38 µg/L, and 0.54 µg/L respectively. The fumigant property of different solvent extracts of *Illicium verum* Hook. f. against *S. zeamais* adults was also reported with the LD_50_ values of the methanol, ethyl acetate, and petroleum ether extracts treatment 7.10, 3.93, and 4.55 mg/l, respectively after 72 h exposure (Li et al., 2013). Essential oils from several aromatic plants also showed fumigant activity. In a study, Devi et al. (2020), reported that essential oils from *Cymbopogon flexuosus* Nees ex Steud. Wats (Poales: Poaceae), *Cymbopogon winterianus* Jowitt ex Bor (Poales: Poaceae), *Cymbopogon martini* Roxb. Wats (Poales: Poaceae), and *Pogostemon cablin* Blanco Benth. (Lamiales: Lamiaceae) exhibited fumigant properties against *S. oryzae*. Essential oils of the Chinese medicinal herb, *Blumea balsamifera* (L.) (Asteraceae) leaves contain 8-cineole, 4-terpineol, and α-terpineol as their main components and were reported to show distinct fumigant toxicity against *S. zeamais* adults (Chu et al., 2013).

The three identified bioactive marker compounds have low molecular weight of 196, 151, and 211 for 6-Hydroxy-4,4,7a-trimethyl-5,6,7,7a-tetrahydrobenzofuran-2(4H)-one (commonly called as loliolide), 1,2-Benzisothiazol-3(2H)-one, and Benzothiazole, 2-(2-hydroxyethylthio)-, respectively, which supports its high volatile property. Dias et al. (2020) reported the antioxidant property of Loliolide isolated from *Sargassum horneri*. The mode of action study was mainly based on the changes in the insect’s behavior when exposed to the fumigant in the fumigation chamber. In the fumigation toxicity experiment, it was observed that immediately after the treatment, the insects start moving rapidly. This may indicate that the extract is acting on the insect’s nervous system like those of organophosphates. Acetylcholine (Ach) is the neurotransmitter that is involved in cholinergic transmission in the brain. AChE enzyme is responsible for the hydrolysis of the neurotransmitter ACh and it is required to rapidly terminate the signaling at neuron junction. Inhibition of the AChE enzyme will cause ACh to bind to the postsynaptic receptor for a longer period causing excessive neuroexcitation. This leads to restlessness, hyperexcitability, tremors, convulsions and paralysis leading to death (Lionetto et al., 2013; Shivanandappa and Rajashekar, 2014). Synthetic insecticides such as organophosphates and carbamates are known to inhibit the acetylcholinesterase enzyme. Therefore, the present study investigated the possible role of the AChE enzyme in the toxicity of the active enriched fraction. The experimental data indicate that the AChE inhibition potential of the enriched fraction was relatively lower than that of the standard. Similar studies have been done by many researchers. In one of the studies, Rajkumar et al., 2019 revealed that the essential oils of *Mentha piperita* L. inhibited the AChE enzyme activity in *S. oryzae*, and *T. castaneum* with LC_50_ values of 29.68%. In a similar study the essential oils of *Ocimum tenuiflorum* (L.) (Lamiales: Lamiaceae) exhibited insecticidal activity *via* inhibiting acetylcholinesterase activity against rice weevil (Bhavya et al., 2018). Coumaran is an active ingredient extracted from *L. camara* which has an inhibitory effect on the insect’s AChE enzyme (Rajashekar et al., 2014).

Antioxidant enzyme system provides the primary defense mechanism of a biological system. Any oxidative imbalance could be detrimental to the normal functioning of many metabolic pathways (FontagneÂ-Dicharry et al., 2014). Free radicals or other reactive oxygen species (ROS) are products of normal metabolism or a result of exposure to any external sources such as rays, ozone, cigarette smoking, certain drugs, pesticides, air pollutants, and industrial chemicals (Sule et al., 2022). Oxidative stress is caused due to the imbalance between the production of free radicals and antioxidant defense in the body. This may lead to chronic and permanent damage to the cell. The bioactive enriched fraction may have caused the production of more free radicals that in turn triggered the system to produce more antioxidant enzymes. This causes a significant increase in the enzymatic activity (SOD, Catalase, GST) as well as non-enzymatic (DPPH) antioxidant systems of both *S. oryzae*, and *T. castaneum* (**Figure 4**). The superoxide dismutase enzyme is responsible for the removal of toxic ROS with the formation of less toxic hydrogen peroxide and oxygen molecules. Catalase enzymes further detoxify the hydrogen peroxide forming non-toxic water and oxygen molecule (Felton and Summers, 1995; El-Amier et al., 2019). Reduced glutathione (GSH) is a non-enzymatic antioxidant that detoxifies the xenobiotics either directly by interacting with reactive oxygen/nitrogen species (ROS and RNS) and electrophiles or by operating as a cofactor for various enzymes (Lushchak, 2012). Glutathione-S-transferase is the enzyme that catalyzes the oxidation of GSH to form oxidized glutathione (GSSH). An increase in GST activity indicated that more of the free radicals have been detoxified. The increased activities of these antioxidant enzymes were dose-dependent (**Figure 4**).

The important characteristic of natural antioxidants is their ability to scavenge free radicals. Proton-radical scavenging action is an important attribute of antioxidants, which is measured by the DPPH radical scavenging assay. DPPH, a protonated radical, has a characteristic absorbance maximum at 517 nm which decreases in the presence of antioxidants due to the scavenging of the proton radical (Yamaguchi 1998). In our study, the enriched fraction was screened for DPPH radical scavenging activity. *In-vitro* experiment results revealed that the enriched fraction isolated from the ethyl acetate extract of *D. indica* leaves had relatively lower free radical scavenging activity when compared with standard ascorbic acid. The percentage scavenging activity was found to increase as we increase the concentration of the fraction (**Figure 4**).

In addition, the present study also showed that the enriched fraction has no significant effect on the seed germination of wheat and green gram. This result is desirable as grain protectants should not have any adverse effect on seed germination. In our previous study, we evaluated the mammalian toxicity of the enriched fraction on both male and female BALB/c mice through acute and sub-acute toxicity and revealed no-observed-adverse-effect level (NOAEL) in the experimental BALB/c mice (Singh et al., 2022). This suggested that the enriched fraction of *D. indica* leaves is significantly safer when compared to other commercially available synthetic fumigants.

## 5 Conclusion

In the present study, for the first time, we have reported the fumigant activity of *D. indica* against *S. oryzae, T. castaneum* and *R. dominica.* The bioactive enriched fraction isolated from ethyl acetate extract of *D. indica* leaves affected the AChE enzyme thereby causing hyperexcitation of the nerve impulse causing paralysis which eventually leads to the death of the insects. The bioactive enriched fraction also causes oxidative imbalance which greatly affects the normal functioning of many metabolic pathways. Three bioactive marker compounds were identified from the enriched fraction, i.e. 6-Hydroxy-4,4,7a-trimethyl-5,6,7,7a-tetrahydrobenzofuran-2(4H)-one (commonly known as loliolide), 1,2-Benzisothiazol-3(2H)-one, and Benzothiazole,2-(2-hydroxyethylthio)-. Our research finding showed that *D. indica* potentially offers a solution to problems associated with health risks, availability, costs, and resistance as in the case of synthetic pesticides. However, further research is needed to identify the bioactive marker compounds, along with its mammalian toxicity to ensure the safety of human and other non-target mammals. Finally, we concluded that *D. indica* could be used as a source of insecticides from plant origin and could be a viable alternative to synthetic insecticides.

## Data availability statement

The original contributions presented in the study are included in the article/**Supplementary Material**. Further inquiries can be directed to the corresponding author.

## Author contributions

KS: conceptualization, validation, formal analysis, investigation, and writing - original draft. AK: manuscript preparation and review. RB: conceptualization, methodology, formal analysis, and review - original draft. YR: project administration, conceptualization, funding acquisition, resources, methodology, supervision, formal analysis, writing - original draft, writing - review and editing, and visualization. All authors contributed to the article and approved the submitted version.
