# Supplementary figures and images for: Insecticidal and biochemical effects of Dillenia indica L. leaves against three major stored grain insect pests
Source: Front Plant Sci. 2023 Feb 20;14:1135946. doi: 10.3389/fpls.2023.1135946 (PMC9986431; doi:10.3389/fpls.2023.1135946)

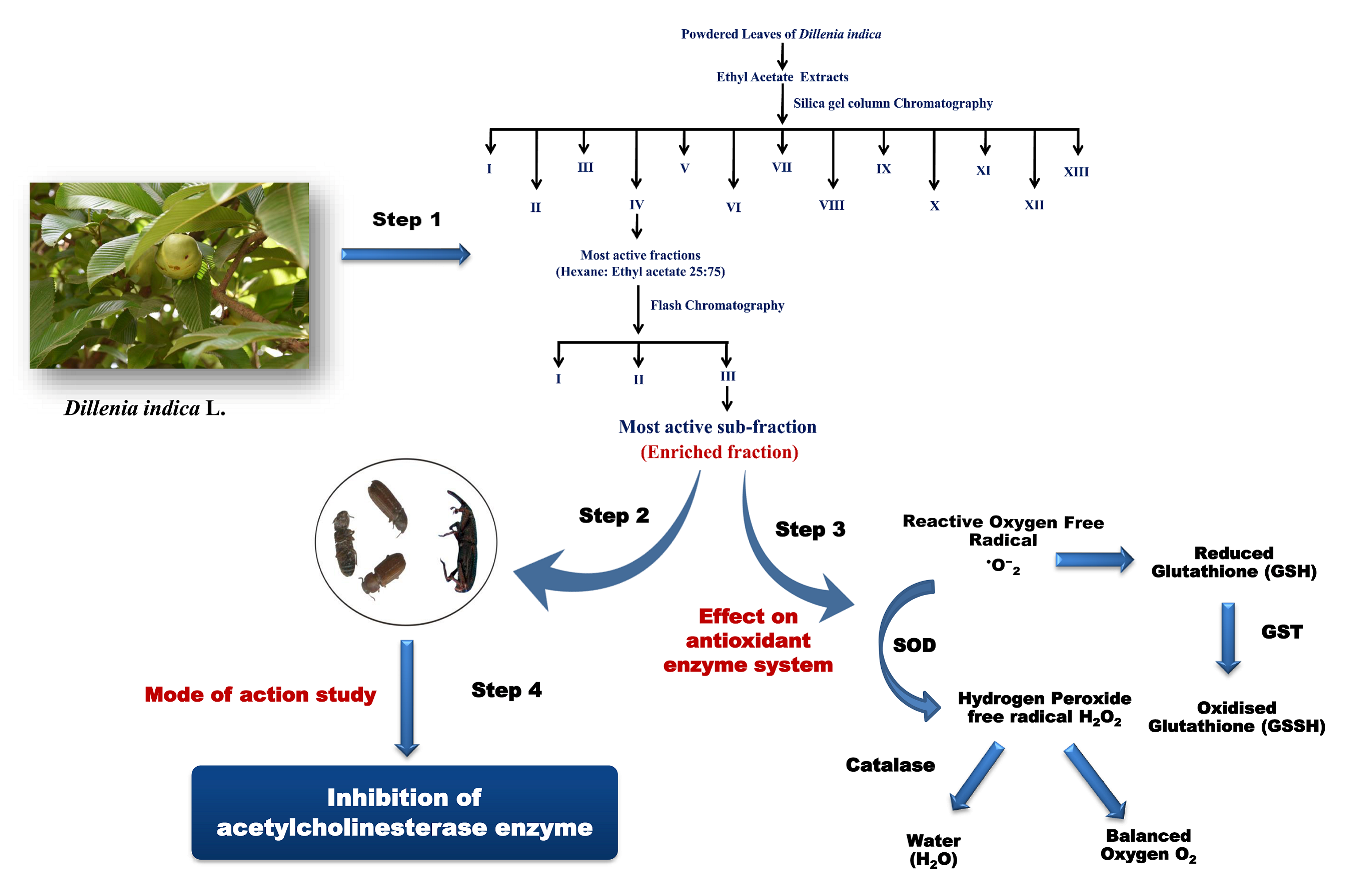

Supplement: Supplementary file 1 [file Image_1.tif]
